# Alleviation of Ovalbumin-Allergic Reactions in Mice by *Eucommia ulmoides* Polysaccharides via Modulation of Intestinal Microbiota

**DOI:** 10.3390/foods14162913

**Published:** 2025-08-21

**Authors:** Xuelei Zhang, Ketong Bi, Chuansheng Zhao, Yuxin Cao, Yuxuan Yang, Jingxuan Jia, Yong Zhang, Dandan Zhai, Yu Yang, Peng Li

**Affiliations:** 1 College of Biological Engineering, Henan University of Technology, Zhengzhou 450001, China; zhangxuelei1020@126.com (X.Z.); biketong6227@163.com (K.B.); 15938250641@163.com (C.Z.); caoyuxin0611@163.com (Y.C.); yongzhang208@haut.edu.cn (Y.Z.); daneszh@haut.edu.cn (D.Z.); 2 School of International Education, Henan University of Technology, Zhengzhou 450001, China; 17518705591@163.com (Y.Y.); jiajingx2004@126.com (J.J.); 3 Institute for Complexity Science, Henan University of Technology, Zhengzhou 450001, China; yangxiangyu1168@haut.edu.cn

**Keywords:** allergy, *Eucommia ulmoides*, polysaccharides, intestinal microbiota

## Abstract

Food allergy represents a prevalent immunological disorder, with current clinical management primarily emphasizing allergen avoidance and emergency pharmacological intervention. *Eucommia ulmoides* polysaccharides, the principal bioactive constituents of the traditional Chinese medicinal plant *Eucommia ulmoides*, have demonstrated anti-inflammatory and antioxidant properties; however, their specific effects on food allergies remain inadequately characterized. A total of thirty-six female BALB/c mice were randomly allocated into three groups (*n* = 12 per group): the control group (CON group, receiving saline treatment), the allergic model group (OVA group, subjected to ovalbumin sensitization), and the intervention group (OVA+PS group, undergoing OVA sensitization followed by *Eucommia ulmoides* polysaccharides administration via gavage). The anti-allergic efficacy of *Eucommia ulmoides* polysaccharides was comprehensively evaluated through clinical allergy symptom scoring, histological and pathological tissue analysis, real-time fluorescence quantitative PCR (qRT-PCR) for the assessment of key gene expression, and 16S rDNA sequencing. The findings indicated the following: (1) The allergy scores in the OVA+PS group were significantly lower than those in the OVA group (*p* < 0.01). Following OVA stimulation, the rectal temperature of mice in the OVA group decreased sharply, whereas the temperature decline in the OVA+PS group was more gradual compared to the model group. (2) The liver, kidney, spleen, and intestinal tissues of mice in the OVA+PS group exhibited normal morphology, consistent with the CON group, which suggests that *Eucommia ulmoides* polysaccharides effectively mitigates the local inflammatory response induced by food allergy. (3) The expression of *NICD* in the spleen of mice in the OVA+PS group was significantly higher than in the OVA group (*p* < 0.05), while the expression of the *Hes1* gene was significantly elevated in the OVA group compared to both the CON and OVA+PS groups (*p* < 0.05). In the OVA group, the expression level of *Gata-3* was significantly elevated compared to both the CON group and the OVA+PS group (*p* < 0.05). Similarly, the expression of *STAT5* in the OVA group was markedly higher than in the other groups (*p* < 0.05). (4) *Eucommia ulmoides* polysaccharides were found to modulate the intestinal microbiota composition in allergic mice, notably increasing the expression abundance of *Enterobacter*, *Oscillibacter*, and *Butyricicoccus*, while decreasing the expression abundance of *Clostridium sensu stricto 1* and *Turicibacter*. (5) There was a correlation between alterations in the intestinal microbiota of mice and the expression of key genes. Specifically, the relative abundance of *Blautia* was negatively correlated with the expression of *NICD* and *Gata-3* genes (*p* < 0.05), and the relative abundance of the *Lachnospiraceae_FCS020_group* was negatively correlated with the expression of the *Hes*1 gene (*p* < 0.05). In conclusion, *Eucommia ulmoides* polysaccharides demonstrate potential in alleviating allergic symptoms, providing a scientific foundation for the development of novel natural anti-allergic functional foods.

## 1. Introduction

The incidence of food allergies has markedly increased in recent years, particularly among children. Food allergies typically arise from an exaggerated immune response to food proteins, leading to a spectrum of clinical manifestations ranging from mild dermatological reactions to severe anaphylactic shock [1,2]. Allergic reactions induced by ovalbumin (OVA) involve the production of immunoglobulin E (IgE) antibodies and the activation of mast cells. IgE antibodies attach to IgE receptors on the surface of mast cells, prompting the release of mediators such as histamine, which subsequently elicit allergic responses [3]. T-helper 2 (Th2) cells, a subset of T cells, are pivotal in OVA-induced allergic reactions. Th2 cells facilitate the production of IgE and sustain allergic responses by secreting cytokines such as interleukin-4 (IL-4), interleukin-5 (IL-5), and interleukin-13 (IL-13) [4].

The development of food allergies is associated with multiple factors, including genetic predispositions, environmental influences, and alterations in the gut microbiota [5,6]. In the OVA-induced allergy model, alterations in the intestinal microbiota are recognized as a critical factor influencing allergic responses. Research indicates that the microbial composition in the intestines of individuals with food allergies significantly differs from that of healthy individuals [7]. Dysbiosis of the intestinal microbiota may exacerbate allergic reactions, whereas the administration of probiotics, such as *Lactobacillus rhamnosus Probio*-M9, has been shown to modulate the microbiota and mitigate allergic symptoms [8]. Furthermore, Fructooligosaccharides (FOS) have been demonstrated to alleviate OVA-induced allergic reactions by modulating the balance of intestinal microbiota and Th17/Treg cells [9]. Bunyavanich et al. [10] conducted an analysis of the intestinal flora in 226 allergic infants, identifying Clostridium and Firmicutes as the most prevalent taxa, and found that the composition of the intestinal flora could significantly ameliorate food allergies. Abdel-Gadir et al. [11] further corroborated the pivotal role of intestinal flora in the regulation of food allergic reactions. Furthermore, a significant increase in the abundance of Firmicutes was observed in children with allergies, whereas a gradual increase in the abundance of *Bifidobacterium* and *Ruminococcus* was noted in children who had developed immune tolerance [12]. This indicates that as allergic symptoms subside, the structure and composition of the intestinal microbiota undergo changes, highlighting the crucial role of gut flora in food allergies.

Certain natural compounds, such as oleuropein, have been shown to effectively prevent and alleviate allergic reactions by enhancing the function of the intestinal epithelial barrier and improving gut microbiota [13]. Similarly, rosmarinic acid has been demonstrated to mitigate OVA-induced allergic symptoms by modulating immune responses and intestinal microbiota [14]. *Eucommia ulmoides* polysaccharides, a principal active component of *Eucommia ulmoides*, exhibits multiple biological functions as a green, safe natural product with no residues or toxic side effects. It has a notable immunomodulatory effect, with studies indicating that *Eucommia ulmoides* polysaccharides can activate the immune system via the TLR4/MyD88/NF-κB signaling pathway, thereby enhancing the body’s resistance to the H1N1 influenza virus [15]. Furthermore, *Eucommia ulmoides* polysaccharides has been shown to enhance the immune function of immunosuppressed mice by modulating the composition of intestinal microbiota [16]. Currently, there is a lack of studies investigating the effects of *Eucommia ulmoides* polysaccharides on food allergies.

In this study, we applied extracted and purified *Eucommia ulmoides* polysaccharides to an OVA-induced mouse allergy model. Experimental groups included a control group, an OVA-sensitized group, and a polysaccharides-treated group. We assessed the impact of *Eucommia ulmoides* polysaccharides on the clinical manifestations of food allergy in mice by evaluating allergy symptom scores, tissue pathology, and changes in intestinal microflora. This study aimed to elucidate the mechanism by which *Eucommia ulmoides* polysaccharides alleviates food allergies, thereby providing a scientific foundation for the comprehensive utilization of *Eucommia ulmoides* leaf resources in mitigating food allergic reactions.

## 2. Materials and Methods

### 2.1. Experimental Materials

*Eucommia ulmoides* polysaccharides were extracted from *Eucommia ulmoides* leaves using ultrasound-assisted hot extraction, with the extraction method comprehensively detailed in the article by Peng Yuqing et al. [17]. Anhydrous ethanol (≥99%), xylene, and glacial acetic acid were procured from Nanjing Jiancheng Co., Ltd. (Nanjing, China). Dimethyl sulfoxide (DMSO) was obtained from Beijing Biolab Technology Co., Ltd. (Beijing, China), and isopropanol (analytical grade) was sourced from Tianjin Zhiyuan Chemical Reagent Co., Ltd. (Tianjin, China). The OVA inducer was acquired from Shanghai Yuanye Biotechnology Co., Ltd. (Shanghai, China). Additionally, a 4% paraformaldehyde fixative, qRT-PCR kit, and reverse transcription kit were supplied by Nanjing Novozyme Biotechnology Co., Ltd. (Nanjing, China). DEPC water and an HE staining kit were purchased from Wuhan Sewell Biotechnology Co., Ltd. (Wuhan, China). PBS buffer and a DNA extraction kit were obtained from Beijing Solebow Technology Co., Ltd. (Beijing, China), while the hematoxylin-eosin staining solution was sourced from Beijing Regen Biotechnology Co., Ltd. (Beijing, China). The CT adjuvant was procured from Sigma (St. Louis, SM, USA).

### 2.2. Experimental Design

**Figure 1:**
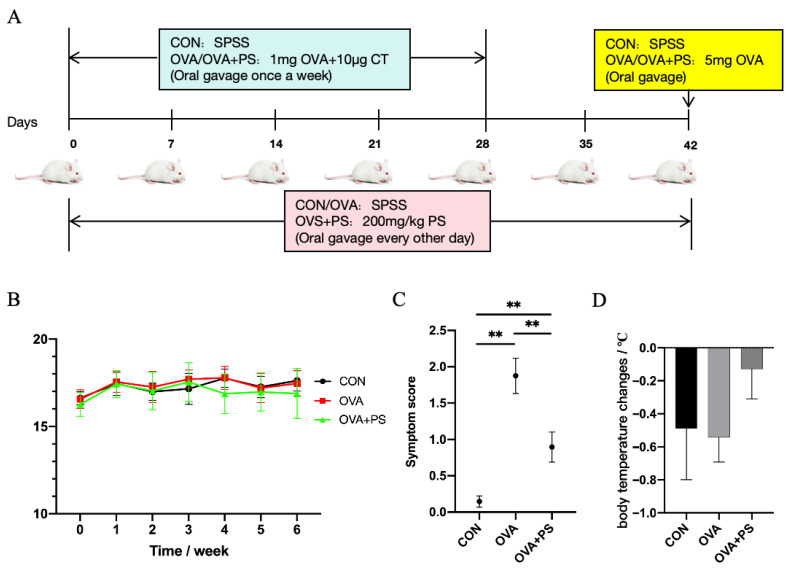
(**A**) Schematic design of the experiment; (**B**) weight change in experimental mice; (**C**) symptom score; (**D**) body temperature changes. (*n* = 8). ** *p* < 0.01.

In this study, BALB/c mice (production license number: SCXK (Henan) 2020-0005) were utilized in accordance with the breeding standards set by the Animal Experiment Center of Henan University of Technology. The experimental design is depicted in Figure 1A. A total of 36 female BALB/c mice were randomly assigned into three groups, each comprising 12 mice: the blank control group (CON group), the allergy model group (OVA group), and the polysaccharides treatment group (OVA+PS group). Mice in the control group received normal saline via gavage, while those in the OVA and OVA+PS groups were administered 1 mg of OVA orally on days 0, 7, 14, 21, and 28. Additionally, mice in the OVA+PS group were gavaged with 200 mg/kg of *Eucommia ulmoides* polysaccharides every other day throughout the experiment. On the 42nd day, a high-dose challenge was conducted. Mice in both the OVA and OVA+PS groups were gavaged with 5 mg of OVA, whereas the control group received an equivalent volume of normal saline. Subsequently, the rectal temperature of each mouse was measured and recorded using a mouse rectal thermometer one hour post-gavage. Following slaughter, tissue samples, including mesenteric lymph nodes, liver, kidney, spleen, and intestine, were collected. The body weights of the mice were measured and recorded weekly throughout the experiment. All animal experimental procedures involving terminal endpoints were conducted in strict accordance with the principles of Replacement, Reduction, and Refinement (the 3Rs) to minimize animal use and suffering.

### 2.3. Scoring of Mouse Allergy Symptoms

The scoring criteria are detailed in Table 1. Scoring was conducted by eight individuals independently, adhering strictly to the established criteria without any interaction or communication among them. The resulting scores underwent statistical analysis.

### 2.4. Histomorphological and Pathological Observations

Tissue samples from the liver, kidney, spleen, and intestine of each group were fixed in a 4% formaldehyde solution. Subsequently, the samples underwent dehydration and clearing using ethanol and xylene, followed by paraffin infiltration and embedding in molten paraffin. Once the wax blocks were prepared, the samples were sectioned. After dewaxing, the sections were stained with hematoxylin–eosin. Images of the stained sections were captured using ECHO software (ECHO LABORATORIES, AZ, USA) with an RVL-100-G upright and inverted integrated fluorescence microscope. Histopathological observation and analysis were performed on the liver, kidney, and spleen sections. The villus height and crypt depth were quantified in the intestinal sections, and the villus-to-crypt ratio was subsequently determined.

### 2.5. Real-Time Fluorescence Quantitative Polymerase Chain Reaction (qRT-PCR)

Approximately 0.05 g of tissue samples from the jejunum, mesenteric lymph nodes, and spleen were collected, and total RNA was extracted utilizing the Vazyme FreeZol Reagent kit (Novozyme, Nanjing, China). The extraction procedure was conducted in accordance with the manufacturer’s protocol. The tissue samples were sectioned and placed into grinding centrifuge tubes. Grinding steel beads and 500 μL of FreeZol Reagent were added, and the mixture was subjected to a pre-cooled centrifugal freezing grinder until complete lysis was achieved. The steel beads were then removed, and the resulting slurry was transferred to a 1.5 mL centrifuge tube, where it was allowed to stand at room temperature for 5 min. The sample was centrifuged at 11,200 rpm and 4 °C for 5 min, after which the supernatant was carefully transferred to a new 1.5 mL centrifuge tube. Subsequently, 100 μL of Dilution Buffer was added to the lysate, the tube was securely capped, vortexed to mix thoroughly, and left to stand at room temperature for an additional 5 min. Centrifuge the sample at 11,200 rpm and 4 °C for 15 min to precipitate impurities. Subsequently, approximately 500 μL of the supernatant should be transferred into a new centrifuge tube. Add an equivalent volume of isopropanol, mix by gentle inversion, and allow the mixture to stand at room temperature for 10 min. Centrifuge again at 11,200 rpm and 4 °C for 10 min, discard the supernatant, and retain the precipitate. Introduce 1 mL of 75% ethanol to resuspend the precipitate, then centrifuge at 9100 rpm and 4 °C for 3 min. Discard the supernatant and retain the precipitate. Repeat the initial two steps, discarding the supernatant thereafter. Allow the precipitate to dry at room temperature, then dissolve it in 20–100 μL of RNase-free double-distilled water (ddH_2_O) to obtain RNA. Utilize RNase-free ddH_2_O as a blank to assess the purity and concentration of the total RNA.

Following the instructions provided by the reverse transcription kit (Novozyme, Nanjing, China), the RNA samples were reverse transcribed, encompassing two steps: genomic DNA removal and the reverse transcription reaction. The genomic DNA removal program was conducted at 37 °C for 30 s, while the reverse transcription program was set at 50 °C for 5 min, followed by 85 °C for 5 s, and then stored at 4° C. Reverse transcription quantitative polymerase chain reaction (RT-qPCR) was conducted in accordance with the protocol provided by the SYBR Green Premix Pro Taq HS qPCR Kit. Details of the reaction system are presented in Table 2. The thermal cycling conditions were as follows: initial denaturation at 95 °C for 30 s; followed by 40 cycles of denaturation at 95 °C for 5 s and annealing/extension at 60 °C for 30 s; and a final dissociation step at 95 °C for 15 s, 60 °C for 1 min, and 95 °C for 15 s. The relative expression levels of the target gene mRNA were quantified using β-actin as the internal control gene. The relative gene expression was calculated using the 2^−ΔΔCt^ method, based on the cycle threshold (Ct) values of both the reference and target genes. The primer sequences utilized in the study are listed in Table 3.

### 2.6. 16S rDNA Sequencing

#### 2.6.1. DNA Extraction and PCR Amplification

Genomic DNA was extracted from samples using the MagPure Soil DNA LQ Kit (Magan) following the manufacturer’s instructions. The concentration and purity of the extracted DNA were assessed using a NanoDrop 2000 spectrophotometer (Thermo Fisher Scientific, Rockford, IL, USA) and agarose gel electrophoresis. The DNA samples were subsequently stored at −20° C. For the amplification of bacterial 16S rRNA genes, the extracted genomic DNA served as a template, and specific primers with barcodes, along with the Takara Ex Taq high-fidelity enzyme, were employed in the PCR amplification process. Universal primers 343F (5′-TACGGRAGGCAGCAG-3′) and 798R (5′-AGGGTATCTAATCCT-3′) were employed to amplify the V3-V4 (or V4-V5) variable regions of the 16S rRNA gene for the purpose of bacterial diversity analysis.

#### 2.6.2. Library Construction and Sequencing

The resulting PCR amplification product was initially detected via agarose gel electrophoresis. Subsequently, the product underwent purification using AMPure XP beads and was utilized as a template for a second round of PCR amplification following purification. The second-round PCR product was further purified using magnetic beads to facilitate Qubit quantification, after which its concentration was adjusted for sequencing. Sequencing was conducted on the Illumina NovaSeq 6000 platform, yielding 250 bp paired-end reads. This sequencing process was carried out by Shanghai Ouyi Biotechnology Co., Ltd. (Shanghai, China).

#### 2.6.3. Bioinformatics Analysis

The initial data were obtained in FASTQ format. Post-download, the raw data sequences were processed using Cutadapt (v5.1) software to remove the primer sequences. Subsequently, DADA2 was employed to conduct quality control analyses, including quality filtering, noise reduction, splicing, and chimera removal, on the qualified paired-end raw data from the preceding step, following the default parameters of QIIME 2 (version 2020.11). This process yielded representative sequences and ASV abundance tables. Representative sequences for each ASV were selected using the QIIME 2 (version 2020.11) software package, after which all representative sequences were compared and annotated using the Silva database (version 138). Species comparison annotation was performed utilizing the default parameters of the q2-feature-classifier. QIIME 2 (version 2020.11) was also utilized for alpha and beta diversity analyses. Alpha diversity was assessed using metrics such as the Chao1 index and the Shannon index. For beta diversity evaluation, the unweighted UniFrac distance matrix was calculated using R and employed in unweighted UniFrac principal coordinate analysis (PCoA). Differential analysis was conducted using the Kruskal–Wallis statistical algorithm within the R package, while LEfSe was applied to perform differential analysis of species abundance profiles.

### 2.7. Data Analysis

The experimental results were mainly analyzed by ANOVA using GraphPad Prism (v8.0). *p* < 0.01 was considered extremely significant, and *p* < 0.05 was considered significant.

## 3. Results

### 3.1. Effect of Eucommia ulmoides Polysaccharides on Allergic Symptoms in Mice

As illustrated in Figure 1B, there was no statistically significant difference in the weight of mice across the groups (*p* > 0.05). Figure 1C demonstrates that the allergy score of the OVA group was significantly elevated compared to both the CON group and the OVA+PS group (*p* < 0.01), while the allergy score of the OVA+PS group was significantly higher than that of the control group (*p* < 0.01). Following OVA stimulation, the rectal temperature of mice in the OVA group decreased sharply, whereas the decline in temperature in the OVA+PS group was more gradual compared to the model group, as depicted in Figure 1D.

### 3.2. Histopathological Analysis

**Figure 2:**
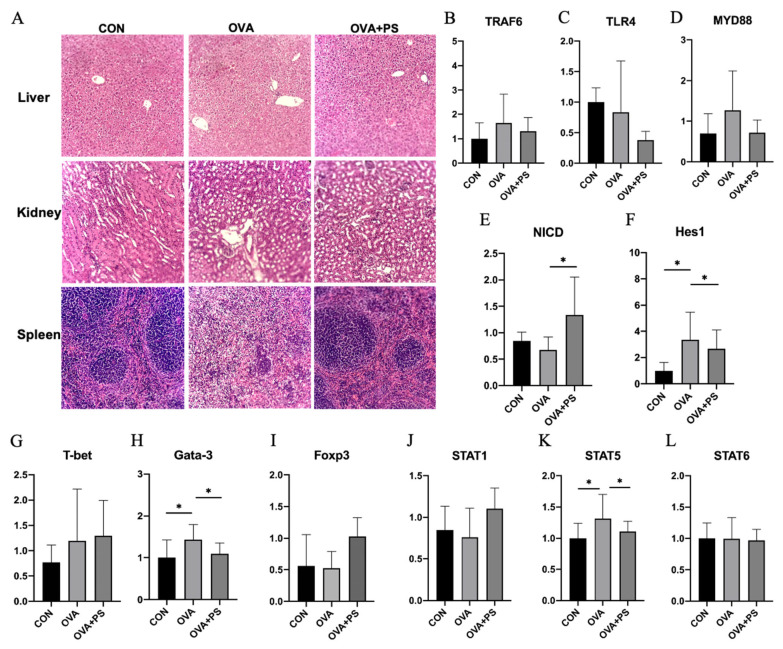
Histopathological analysis of liver, kidney, and spleen tissues and gene expression in the spleen. (**A**) H&E staining of liver, kidney, and spleen sections (*n* = 5); (**B**–**L**) relative mRNA levels of *TRAF6*, *TLR4*, *MYD88*, *NICD*, *Hes1*, *T-bet*, *Gata-3*, *Foxp3*, *STAT1*, *STAT5*, and *STAT6* in the spleen. The relative mRNA expression levels of genes detected by quantitative real-time PCR. Data are presented as mean ± SD (*n* = 3). * (*p* < 0.05) indicates the significant difference compared with every group.

Histological examination of the liver, kidney, and spleen tissues, stained with hematoxylin and eosin (HE), revealed the pathological features presented in Figure 2A. In comparison to the CON group, the OVA group exhibited lymphocyte infiltration and inflammatory cell distribution within the liver, kidney, and spleen. Conversely, the tissues in the OVA+PS group maintained normal morphology, with no apparent inflammation. These findings indicate that *Eucommia ulmoides* polysaccharides effectively mitigates the local inflammatory response induced by food allergy.

### 3.3. Effect of Eucommia ulmoides Polysaccharides on the Expression of Kidney-Related Genes in Mice

The expression of genes associated with splenic inflammation, key components of the Notch signaling pathway, and critical genes involved in Th1/Th2 cell differentiation were analyzed using quantitative reverse transcription polymerase chain reaction (qRT-PCR). The expression profiles of inflammatory genes are illustrated in Figure 2B–D. No statistically significant differences were observed in the expression levels of the *TRAF6*, *TLR4*, and *MYD88* genes across the experimental groups (*p* > 0.05). In contrast, analysis of the Notch signaling pathway revealed that the expression of *NICD* was significantly elevated in the spleens of mice within the OVA+PS group compared to the OVA group (*p* < 0.05), as depicted in Figure 2E. Additionally, the expression of the *Hes1* gene was significantly higher in the OVA group than in both the CON and OVA+PS groups (*p* < 0.05), as shown in Figure 2F. Concerning Th1/Th2 cell differentiation, the expression level of *Gata-3* was significantly increased in the OVA group relative to the CON and OVA+PS groups (*p* < 0.05), while no significant differences were detected in the expression levels of *T-bet* and *Foxp3* among the groups (*p* > 0.05), as presented in Figure 2G,I. In the OVA group, the expression of *STAT5* was markedly elevated compared to the other groups (*p* < 0.05) (Figure 2K), while no significant differences were observed in the expression levels of *STAT1* and *STAT6* across the groups (*p* > 0.05) (Figure 2J,L).

### 3.4. Analysis of Mouse Intestinal Tissue Morphology

**Figure 3:**
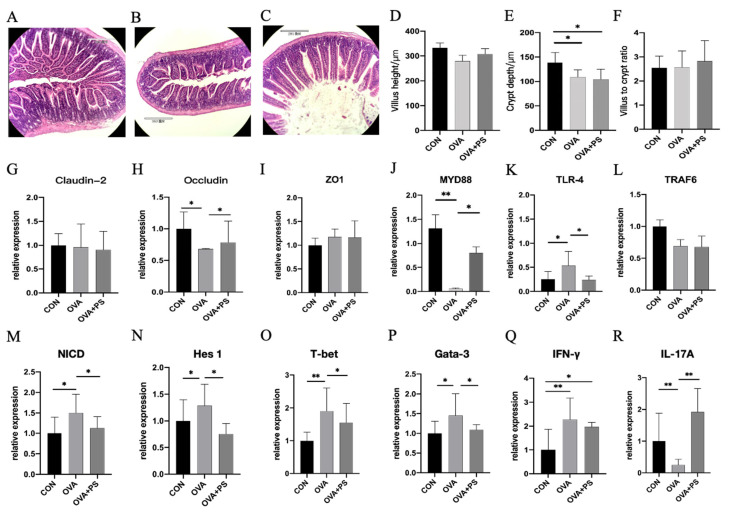
(**A**) Jejunuml intestinal morphology of CON group (*n* = 5); (**B**) Jejunuml intestinal morphology of OVA group (*n* = 5); (**C**) Jejunuml intestinal morphology of OVA+PS group (*n* = 5); (**D**) Villus height of jejunum; (**E**) Crypt depth of jejunum; (**F**) Villus height to Crypt depth (V:C) ratio; (**G**–**L**) Relative mRNA levels of *Claudin-2*, *Occludin*, *ZO1*, *MYD88*, *TLR-4* and *TRAF6* in jejunum. (**M**–**R**) Relative mRNA levels of *NICD*, *Hes1*, *T-bet*, *Gata-3*, *IFN-γ*, and *IL-17A* in mesenteric lymph nodes. The relative mRNA expression levels of genes detected by quantitative real-time PCR. Data are presented as mean ± SD (*n* = 3). * (*p* < 0.05) and ** (*p* < 0.01) indicate the significant difference compared with every group.

Figure 3A illustrates that the epithelial tissue structure in the CON group was intact, with closely arranged epithelial cells, intact glands, and an absence of lesions. Conversely, in the OVA group, the muscle layer was thinner, the mucosal gap was enlarged, and the integrity of the epithelial tissue was compromised (Figure 3B). As depicted in Figure 3C, the OVA+PS group exhibited a reduced distance between intestinal glands in the lamina propria, thickening of the lamina propria, a significant increase in intestinal cells, and a notable improvement in intestinal inflammation. The crypt depth in the CON group was significantly greater than that in both the OVA and OVA+PS groups (*p* < 0.05) (Figure 3E), whereas no significant differences were found in villus height and the villus/crypt ratio among the groups (*p* > 0.05) (Figure 3D,F).

### 3.5. Effect of Eucommia ulmoides Polysaccharides on the Expression of Genes Related to the Intestine and Mesenteric Lymph Nodes of Mice

As illustrated in Figure 3H, the expression level of the *Occludin* gene in the jejunum of the OVA group was significantly elevated compared to both the CON group and the OVA+PS group (*p* < 0.05). No significant differences were observed in the expression levels of the *Claudin-2* and *ZO1* genes among the groups (*p* > 0.05) (Figure 3G,I). The expression of the *MYD88* gene in the OVA group was significantly or extremely significantly reduced relative to the CON and OVA+PS groups (*p* < 0.05 or *p* < 0.01) (Figure 3J). The *TLR-4* gene expression level in the OVA group was significantly higher than in the other two groups (*p* < 0.05) (Figure 3K). There was no significant difference in the expression level of the *TRAF6* gene in the jejunum across the groups (*p* > 0.05). Regarding gene expression in the mesenteric lymph nodes, the expression levels of *NICD*, *Hes-1*, *Gata-3*, and *T-bet* in the OVA group were significantly higher than those in the CON and OVA+PS groups (*p* < 0.05 or *p* < 0.01) (Figure 3M–P). As depicted in Figure 3Q, the expression level of *IFN-γ* in the CON group was significantly or extremely significantly higher than in the OVA and OVA+PS groups (*p* < 0.05 or *p* < 0.01). The expression level of *IL-17A* in the OVA group was significantly lower than that in both the CON and OVA+PS groups (*p* < 0.01).

### 3.6. Effects of Eucommia ulmoides Polysaccharides on the Structure and Function of Mouse Intestinal Microflora

#### 3.6.1. Diversity Analysis

**Figure 4:**
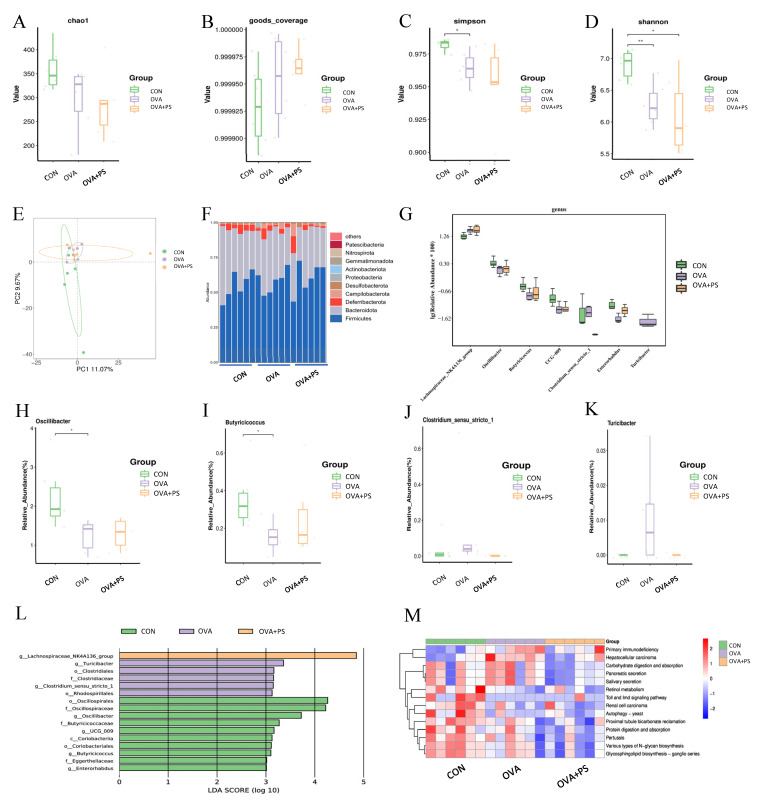
Analysis of intestinal flora structure and function (*n* = 6). (**A**–**D**) Alpha diversity analysis-Chao1 index, Good’s coverage index, Simpson index, and Shannon index; (**E**) beta diversity analysis-PCoA Analysis; (**F**) comparison of the relative abundance of top 10 phyla; (**G**) the different phylum compositions of the experimental group; (**H**–**K**) the expression abundance of differential bacterial genera; (**L**) LEfSe analysis; (**M**) heat map of KEGG by PICRUSt analysis. * (*p* < 0.05) and ** (*p* < 0.01) indicate the significant difference compared with every group.

Alpha diversity analysis indicated no significant differences in the Chao1 index and Good’s coverage index among the groups (*p* > 0.05) (Figure 4A,B). The Simpson index in the CON group was significantly higher compared to the OVA group (*p* < 0.05), while the Shannon index in the CON group was significantly or highly significantly higher than those in the OVA+PS and OVA groups (*p* < 0.05 or *p* < 0.01). Beta diversity analysis of the microbial community structure, visualized using Principal Coordinates Analysis (PCoA), revealed that the PC1 and PC2 factors accounted for 11.07% and 9.6% of the variance, respectively. These findings demonstrate that the microbial communities among the three groups were significantly distinct.

#### 3.6.2. Analysis of Intestinal Bacterial Composition

The composition of intestinal microbiota across various treatment groups was examined at the phylum level. As illustrated in Figure 4F, Firmicutes and Bacteroidota constituted over 90% of the total intestinal microbiota, serving as the predominant components across the different treatment groups. At the genus level, seven distinct genera were identified, including *Lachnospiraceae_NK4A136_group*, *Oscillibacter*, *Butyricicoccus*, *UCG-009*, *Clostridium_sensu_stricto_1*, *Enterorhabdus*, and *Turicibacter* (Figure 4G). Notably, the abundance of *Oscillibacter* and *Butyricicoccus* was significantly higher in the CON group compared to the OVA group (*p* < 0.05), with no significant difference observed in comparison to the OVA+PS group (*p* > 0.05) (Figure 4H,I). Relative to the OVA group, *Eucommia ulmoides* polysaccharides treatment resulted in a downregulation of *Clostridium sensu stricto 1* and *Turicibacter* abundance (Figure 4J,K), while upregulating the abundance of *Enterorhabdus* (Figure 4G). As illustrated in Figure 4L, the *Lachnospiraceae_NK4A136_group* emerged as the predominant genus within the OVA+PS group. In contrast, the OVA group was characterized by the dominance of genera such as *Turicibacter*, *Clostridiales*, *Clostridiaceae*, *Clostridium_seneu_stricto_1*, and *Rhodospirillales*. The introduction of polysaccharides led to an increase in dominant genera, including *Oscillospirales*, *Oscillospiraceae*, *Oscillibacter*, *Butyricicoccaceae*, *UCG_009*, *Coriobacterila*, *Coriobacteriales*, *Butyricicoccus*, *Eggerthellaceae*, and *Enterorhabdus*.

#### 3.6.3. PICRUSt Function Prediction

According to the PICRUSt functional prediction analysis presented in Figure 4M, the incorporation of polysaccharides modulates several biological processes, such as primary immunodeficiency, various types of N-glycan biosynthesis, glycosphingolipid biosynthesis, proximal tubule bicarbonate reclamation, protein digestion and absorption, retinol metabolism, carbohydrate digestion and absorption, and pancreatic secretion. These functions are crucial for maintaining the normal physiological operations of animals.

### 3.7. Correlation Analysis Between Key Gene Expression and Mouse Intestinal Microorganisms

**Figure 5:**
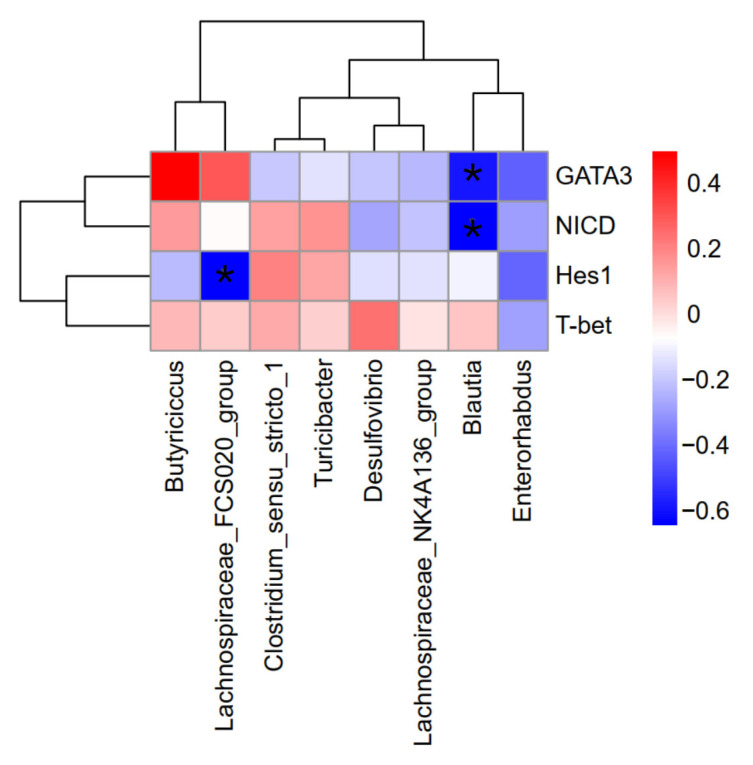
Correlation analysis of key genes and differential bacterial species (*n* = 6). * (*p* < 0.05) indicates a significant correlation.

A correlation analysis was conducted to examine the relationship between differentially expressed genes in mesenteric lymph nodes and differential bacterial species. This analysis aimed to further elucidate the impact of *Eucommia ulmoides* polysaccharides on the correlation between alterations in mouse intestinal microbiota and key gene expression. As illustrated in Figure 5, the relative abundance of Blautia was found to be negatively correlated with the expression of *NICD* and *GATA3* genes (*p* < 0.05). Additionally, the relative abundance of the *Lachnospiraceae_FCS020_group* exhibited a negative correlation with the expression of the *Hes1* gene (*p* < 0.05).

## 4. Discussion

### 4.1. The Influence of Eucommia ulmoides Polysaccharides on Allergic Symptoms in Mice

The global incidence of allergic symptoms is on the rise, emerging as a significant factor impacting human health. Allergic reactions typically result from the immune system’s exaggerated response to benign environmental substances, manifesting in symptoms such as rhinitis, asthma, and eczema. These conditions not only diminish the quality of life for affected individuals but can also precipitate more severe health complications [18]. Within the scope of allergic symptomatology, the relationship between allergies and body weight is a pertinent area of investigation. Research indicates a correlation between allergic symptoms and alterations in pediatric body weight; for instance, food allergies may lead to inadequate nutritional intake in children, thereby impeding their normal growth and development [19]. Moreover, the severity of allergic symptoms may indirectly influence body weight by affecting appetite and metabolic rate. In the present study, due to the relatively low baseline weight of the mice, no significant differences in body weight were observed across the experimental groups. The Allergy Control Score, a novel symptom-drug scoring system, has been validated as an effective instrument for evaluating allergy severity and accurately reflecting the overall intensity of allergic reactions [18]. In this study, the score for the OVA group was significantly higher than that of the CON group, demonstrating the successful establishment of the allergy model in this experiment. The allergy score for the polysaccharides group was notably lower than that of the model group, yet significantly higher than that of the control group, suggesting that polysaccharides exert a comparable ameliorative or therapeutic effect on allergic symptoms. Furthermore, allergic symptoms may influence rectal temperature. Allergic reactions are frequently accompanied by inflammatory responses, which can result in elevated body temperature. As a potential indicator of inflammatory response, this warrants further investigation and consideration. The impact of allergic symptoms on body weight, clinical scores, and rectal temperature represents a complex and multidimensional issue that necessitates multidisciplinary research to thoroughly elucidate its mechanisms and effects. Such studies not only enhance the diagnosis and management of allergic symptoms but also provide a scientific foundation for the development of more effective treatment strategies.

### 4.2. The Influence of Polysaccharides on Histopathological Analysis and Expression of Key Genes in Mice

Food allergy is a chronic allergic condition that currently lacks a definitive cure and can lead to serious complications, including mortality. Consequently, we conducted a histopathological analysis of the liver, kidney, and spleen in allergic mice. Histopathological examination revealed that ovalbumin (OVA) exposure resulted in significant immune cell infiltration and the presence of inflammatory cells [20]. In this study, we also observed lymphocyte infiltration and inflammatory cell distribution in the liver, kidney, and spleen of mice in the OVA model group. However, following the administration of *Eucommia ulmoides* polysaccharides, there was a reduction in lymphocyte infiltration and inflammatory cell distribution in these organs, with no evident signs of inflammation. This study demonstrates that *Eucommia ulmoides* polysaccharides effectively mitigates the local inflammatory response associated with food allergies.

In allergic reactions, the activation of immune cells and the release of cytokines are critical processes. NICD and Hes1, components of the Notch signaling pathway, play significant roles in the immune system by regulating cell differentiation, proliferation, and apoptosis. NICD, the intracellular domain of the Notch receptor, is released upon ligand activation and subsequently enters the cell nucleus, where it binds to transcription factors to regulate the expression of target genes. The findings of this study indicate that polysaccharides can significantly enhance *NICD* expression to modulate allergic reactions while concurrently reducing *Hes1* gene expression. Further research is warranted to elucidate the specific mechanisms of action of NICD and Hes1 in allergic reactions, with the aim of identifying novel targets and strategies for the treatment of allergic diseases. Additionally, T-bet and Gata-3 are crucial transcription factors involved in the differentiation of Th1 and Th2 cells. Research has demonstrated that the upregulation of T-bet is linked to the Th1 immune response, whereas the upregulation of Gata-3 is linked to the Th2 immune response [21,22]. In models of allergic asthma, the overexpression of Gata-3 has been shown to increase susceptibility to allergic airway inflammation [23]. This study indicates that polysaccharides can downregulate Gata-3 expression, thereby exerting a modulatory effect on allergic reactions. Foxp3, recognized as a key transcription factor of regulatory T cells (Tregs), is typically associated with enhanced immunosuppression and diminished inflammation when its expression is elevated. In the context of allergic asthma models, an increase in *Foxp3* expression correlates with reduced airway inflammation [24]. Consistent with previous findings, this study observed that polysaccharides tend to upregulate *Foxp3* expression, contributing to the attenuation of inflammatory responses. Furthermore, STAT proteins, which function as signal transducers and transcriptional activators, play a crucial role in various biological processes, including cell proliferation, differentiation, and immune responses. In allergic reactions, STAT proteins modulate the intensity and duration of inflammatory responses by regulating the activation of immune cells and the expression of inflammatory mediators. A study investigated the role of STAT transcription factors using a rat model of asthma. Employing decoy oligodeoxynucleotides (ODN) specific to STAT1 and STAT3, the study demonstrated a significant reduction in the number of eosinophils and T lymphocytes in bronchoalveolar lavage fluid. Furthermore, there was a notable decrease in the number of CD4^+^ and CD8^+^ lymphocytes in lung tissue, along with a reduction in CD40 protein expression. These findings suggest that the localized application of STAT decoy ODN effectively attenuates allergen-induced cellular inflammatory responses [25].

TRAF6, TLR4, and MYD88 are critical components of the Toll-like receptor signaling pathway, playing a pivotal role in the innate immune response. Allergic reactions are initiated by the immune system’s exaggerated response to typically innocuous substances and are integral to the innate immune response. Research has demonstrated that Toll-like receptor 4 (TLR4) is integral to the pathogenesis of allergic rhinitis, with alterations in its expression and function potentially contributing to the onset and progression of the disease [26]. Furthermore, TLR4 is implicated in the pathophysiological mechanisms of allergic airway inflammation, facilitating the initiation of Th2-type immune responses through the activation of dendritic and other immune cells [27]. TNF receptor-associated factor 6 (TRAF6), functioning as an E3 ubiquitin ligase, is pivotal in signal transduction within the TLR signaling pathway. The interaction between TRAF6 and protein phosphatase PP1-γ enhances TRAF6 activity, thereby augmenting NF-κB-mediated innate immune signaling responses [28]. Myeloid differentiation primary response 88 (MYD88) serves as a crucial adaptor protein in the TLR signaling pathway, responsible for relaying TLR activation signals to downstream signaling molecules. MYD88-dependent pathways are involved in various immune responses, including those against viral and bacterial infections [29]. In the present study, *Eucommia ulmoides* polysaccharides did not exhibit a significant impact on the expression of the TRAF6, TLR4, and MYD88 genes.

It is hypothesized that *Eucommia ulmoides* polysaccharides could influence organ histopathology in murine models of allergy through analogous mechanisms. These polysaccharides may mitigate tissue damage associated with allergic reactions by modulating immune cell activity and cytokine expression. Further empirical investigations are required to substantiate this hypothesis and elucidate the potential therapeutic efficacy of *Eucommia ulmoides* polysaccharides in the management of allergic diseases.

### 4.3. Analysis of Intestinal Histological Morphology and Expression of Key Genes in Intestinal and Mesenteric Lymph Nodes of Mice

Food allergies frequently result in intestinal inflammation, which subsequently causes damage to the intestinal barrier. This barrier serves as the primary defense mechanism against allergens. Maintaining the integrity of the intestinal epithelium is crucial for preventing various inflammatory conditions and is also a focal point in the treatment of numerous immune-related diseases [30]. In this study, mice in the allergic model group exhibited enlarged gaps in the jejunal submucosal layer, partial detachment of mucosal epithelial cells, disorganized glandular structures, and pronounced inflammation. However, upon administration of *Eucommia ulmoides* polysaccharides to the allergic mice, a significant increase in the height of intestinal villi was observed, along with a reduction in crypt depth, an increase in the number of intestinal cells, and marked improvement in intestinal inflammation. These findings suggest that *Eucommia ulmoides* polysaccharides may enhance the integrity of the intestinal barrier and mitigate food allergic reactions. The expression of the *Occludin* gene appears to influence the barrier function of the jejunum, thereby playing a role in allergic responses. Alterations in intestinal barrier function may impact the entry of allergens and the subsequent activation of immune responses. When the jejunal barrier function is compromised due to alterations in the expression of the *Occludin* gene, the intestinal barrier’s effectiveness against allergens is diminished, facilitating the entry of allergens into the body. This can subsequently activate the immune response and potentially trigger an allergic reaction. In the jejunum of mice exhibiting allergic responses, the *MYD88* gene may also play a role in the transmission of immune signals. Upon allergen entry into the jejunum, related immune cells may be activated, initiating an immune response via the MYD88-mediated signaling pathway. Following the administration of lipopolysaccharide (LPS) into the mouse brain, LPS activates TLR-4 and its downstream molecules, such as MyD88, thereby inducing an inflammatory response [31]. In the jejunum of mice experiencing allergic reactions, the *TLR-4* gene may similarly be activated by allergens, interacting with *MYD88* to initiate a cascade of immune and inflammatory responses, thereby influencing the physiological state of the jejunum. These observations indicate that the expression of *Occludin*, *MYD88*, and *TLR-4* genes in the jejunum of mice with allergic reactions is interconnected and collectively contributes to the onset and progression of allergic reactions. Alterations in the expression of the *Occludin* gene may influence the barrier function of the jejunum, facilitating the penetration of allergens. The *MYD88* and *TLR-4* genes are pivotal in immune signal transduction and inflammatory responses, and their interaction may dictate the intensity and progression of allergic reactions.

The Notch signaling pathway promotes Th2 cell differentiation and exacerbates allergic responses by upregulating *Gata-3* expression. Additionally, the Notch signaling pathway can downregulate *T-bet*, a critical Th1 transcription factor, thereby enhancing allergic reactions by inhibiting Th1 responses, promoting Th2 responses, and disrupting the Th1/Th2 balance. The Notch signaling pathway is positively associated with the regulation of food allergies [32]. This study examined the expression levels of *NICD* and *Hes1*, key components of the Notch signaling pathway, in mesenteric lymph nodes and discovered that *Eucommia ulmoides* polysaccharides significantly reduce the expression of *NICD* and *Hes1*. We hypothesize that *Eucommia ulmoides* polysaccharides may inhibit the expression of the Notch receptor and the activity of γ-secretase, thereby suppressing the Notch signaling pathway and mitigating allergic reactions. Furthermore, the interaction between the gut microbiota and the intestinal mucosal immune system plays a crucial regulatory role in the onset and progression of food allergies. The early maturation of the intestinal microbiota in mice can alter the Th2/Th1 balance, enhancing the Th1 cell response. Conversely, dysregulation of the mucosal immune system can shift the Th1/Th2 cytokine balance towards Th2 dominance [33]. Our study demonstrates that *Eucommia ulmoides* polysaccharides significantly downregulate the expression of *Gata-3*, a pivotal gene for Th2 differentiation, while upregulating *T-bet*, a critical gene for Th1 cell differentiation. This modulation inhibits the Th2 immune response, enhances the Th1 immune response, and promotes a balanced Th1/Th2 response, thereby reducing allergic reactions. These findings suggest that *Eucommia ulmoides* polysaccharides may regulate intestinal immunity and alleviate allergic reactions by modulating the expression of key genes within the Notch signaling pathway.

### 4.4. Influence of Eucommia ulmoides Polysaccharides on the Structural and Functional Dynamics of Intestinal Microbiota in Mice

Food allergies have been closely linked to alterations in the intestinal microbiota [34]. Research conducted by Li Xinyue et al. [12] demonstrated significant differences in the composition and structure of the intestinal microbiota between healthy and allergic mice, with the microbiota of healthy mice capable of mitigating allergic symptoms in affected mice. Furthermore, studies have identified the adhesion ability of Candida as a critical factor in its pathogenicity, as this ability initiates the colonization process and facilitates its persistence within the host [35]. Although research on *Enterobacter* remains relatively limited, its role within the intestinal microbiota is significant, potentially influencing intestinal health and disease through interactions with the host immune system. In this study, it was observed that, compared to the OVA group, *Eucommia ulmoides* polysaccharides increased the abundance of *Candida* (*UGC-009*) and *Enterobacter* (*Enterorhabdus*), aligning with the abundance trends observed in the control group. Additionally, *Butyriciccus*, a probiotic known for its ability to regulate intestinal microecological balance, has demonstrated therapeutic effects on intestinal inflammation [36]. The health effects of this compound are primarily mediated through the production of butyrate, a short-chain fatty acid with diverse physiological roles, including the enhancement of intestinal barrier function and the modulation of the immune system [37]. A study demonstrated that supplementation with *Butyriciccus* significantly elevated butyrate levels in the intestines of mice, thereby promoting intestinal health [38]. At the genus level, the abundance of *Butyriciccus* in the OVA group of mice was markedly reduced. However, intervention with *Eucommia ulmoides* polysaccharides restored the proportion of *Butyriciccus* in the intestines of allergic mice to levels comparable to the control group. We hypothesize that *Eucommia ulmoides* polysaccharides may exert an anti-allergic effect in mice by modulating *Butyriciccus* microorganisms. The *Lachnospiraceae_NK4A136_group* genus is known for its roles in promoting mucosal repair and exerting anti-inflammatory effects [39]. The increased abundance of *Lachnospiraceae_NK4A136_group* in the polysaccharides-treated group suggests a reparative effect on intestinal damage induced by allergic reactions. Furthermore, the predominant bacterium in the allergic cohort, *Clostridium seneu stricto 1*, is known to cause conditions such as enteritis [40]. *Eucommia ulmoides* polysaccharides have been shown to mitigate the expression of *Clostridium seneu stricto* 1, suggesting that modulation of gut microbiota composition may be a crucial mechanism by which *Eucommia ulmoides* polysaccharides prevent food allergies.

### 4.5. Correlation Analysis Between Differential Gene Expression and Intestinal Bacterial Species in Mice

The relationship between intestinal microorganisms and key genes was examined by correlating the expression of key differential genes with differential bacterial species in the murine intestine. The findings indicated that the relative abundance of Blautia was inversely correlated with the expression of *NICD* and *Gata-3* genes. *Eucommia ulmoides* polysaccharides enhanced the abundance of *Blautia* by downregulating *NICD* and *Gata-3* expression. *Blautia* is an anaerobic genus with probiotic properties, commonly found in the mammalian gut, and is known for its beneficial effects. It is capable of biotransformation, regulation of host health, and alleviation of metabolic syndrome [41]. The composition and dynamics of *Blautia* populations within the intestinal microbiome are associated with various physiological states of the host. This genus has been implicated in biotransformation processes in conjunction with other intestinal microorganisms [42,43,44]. *Lachnospiraceae*, a significant group within the human intestinal microbiome, plays a crucial role in maintaining host health through several mechanisms, including the production of short-chain fatty acids (SCFAs) and the modulation of immune responses. Research conducted by Vacca et al. [45] demonstrated substantial inter- and intraspecific diversity among *Lachnospiraceae* strains, which may influence their capacity to produce SCFAs, convert bile acids, and resist pathogen colonization. In the present study, a negative correlation was observed between the relative abundance of the *Lachnospiraceae_FCS020_group* and *Hes1* gene expression.

## 5. Conclusions

*Eucommia ulmoides* polysaccharides has been shown to effectively alleviate allergic symptoms and address local inflammatory reactions induced by food allergies in murine models. The mechanism by which *Eucommia ulmoides* polysaccharides mitigates allergic responses may involve the modulation of key gene expressions, such as *NICD* and *Hes1* within the Notch signaling pathway, as well as *T-bet*, *Gata-3*, and *Foxp3* in the differentiation of Th1/Th2 cells. Furthermore, *Eucommia ulmoides* polysaccharides influences the composition of the intestinal microbiota in allergic mice, with the abundance of specific intestinal microorganisms, such as *Blautia* and the *Lachnospiraceae_FCS020_group*, being associated with the expression levels of critical genes *NICD*, *Gata-3*, and *Hes1*.

## Figures and Tables

**Table 1:** Mouse allergy symptom scoring table.

Symptom	Score
Asymptomatic	0
Scratching ears and nose	1
Swelling around the eyes and nose; decreased activity; shortness of breath	2
Increased breathing rate; wheezing; difficulty breathing; hair loss, redness, and swelling around the mouth and nose; rash on the tail	3
Unresponsiveness to stimulation; muscle contractions; cramps	4
Convulsion; shock; death	5

**Table 2:** RT-qPCR reaction system.

Component	Volume
SYBR qPCR Master Mix	5.0 μL
F Primer	0.2 μL
R Primer	0.2 μL
Template	2.0 μL
ddH_2_O	2.6 μL

**Table 3:** Primer design.

Gene	Primer Sequences (Forward)	Primer Sequences (Reverse)
*TRAF6*	AAAGCGAGAGATTCTTTCCCTG	ACTGGGGACAATTCACTAGAGC
*TLR4*	ATGGCATGGCTTACACCACC	GAGGCCAATTTTGTCTCCACA
*MYD88*	TCATGTTCTCCATACCCTTGGT	AAACTGCGAGTGGGGTCAG
*Claudin-2*	CCTCGCTGGCTTGTATTATCTCTG	GAGTAGAAGTCCCGAAGGATG
*Occludin*	TTGAAAGTCCACCTCCTTACAGA	CCGGATAAAAAGAGTACGCTGG
*ZO1*	ACCACCAACCCGAGAAGAC	CAGGAGTCATGGACGCACA
*IFN-γ*	GCGTCTTGGTTTTGCAGCTC	ACCGTCCTTTTGCCAGTTCC
*IL-17A*	CACTTCACAAGTCGGAGGCT	TCTGACAGTGCATCATCGCT
*NICD*	CCAACTGAGGACAGACGGCA	GGGATCAGAGGCCACATAGC
*Hes1*	CGAGTGCATGAACGAGGTGA	ATCTGGGTCATGCAGTTGGC
*T-bet*	CGTTTCTACCCCGACCTTCC	ATGCTCACAGCTCGGAACTC
*Gata-3*	AAGCTCAGTATCCGCTGACG	GATACCTCTGCACCGTAGCC
*Foxp3*	CCCATCCCCAGGAGTCTTG	ACCATGACTAGGGGCACTGTA
*STAT1*	TCACAGTGGTTCGAGCTTCAG	GCAAACGAGACATCATAGGCA
*STAT5*	CTCTGTGGGGCCTAATTTCCA	TCCTGGGGATTATCCAAGTCAAT

## Data Availability

The original contributions presented in the study are included in the article, further inquiries can be directed to the corresponding author.
